# Genome-wide identification and expression pattern analysis of the chloride channel gene family in potato (*Solanum tuberosum* L.) under salt stress

**DOI:** 10.3389/fpls.2025.1703856

**Published:** 2025-10-22

**Authors:** Huiling Gong, Hang Wang, Jie Chen, Leonce Dusengemungu, Zaiping Feng

**Affiliations:** ^1^ College of Life Sciences and Engineering, Lanzhou University of Technology, Lanzhou, China; ^2^ African Agricultural Technology Foundation, Rwanda Agriculture Board (RAB) Rubirizi Station, Kigali, Rwanda

**Keywords:** potato, CLC, salt stress, expression analysis, qRT-PCR

## Abstract

**Introduction:**

Chloride channel (*CLC*) proteins are crucial anion channels that play a vital role in plant adaptation to abiotic stresses. Potato (*Solanum tuberosum* L.) is a major global staple crop; however, the *CLC* gene family in potato (*StCLC*) remains poorly characterized, and its specific functions in salt tolerance are unclear. This study aimed to systematically identify and characterize the *StCLC* gene family and analyze its expression patterns under salt stress.

**Methods:**

Using bioinformatics methods based on the potato genome, transcriptome, and qRT-PCR data, we analyzed the protein structures, physicochemical properties, phylogenetic relationships, gene structures, conserved domains, chromosomal locations, collinearity, GO annotations, and promoter *cis*-acting elements of *StCLC* members.

**Results:**

Seven *StCLC* genes (*StCLC1–7*) were identified and unevenly distributed across four chromosomes. Based on protein structures and phylogenetic relationships with *Arabidopsis thaliana CLCs*, the seven *StCLCs* were classified into three clusters. Gene structure analysis revealed that *StCLC* genes contain 6–9 exons, and Motifs 6, 7, 8, and 9 were conserved across all seven *StCLC* proteins, suggesting their functional importance. Collinearity analysis indicated that the *StCLC* family has its own collinear genes and shares a close evolutionary relationship with the tomato (*Solanum lycopersicum* L) *CLC* family. GO annotation indicated that *CLCs* are primarily involved in chloride ion transport. Thirty-five *cis*-acting regulatory elements were identified in the promoter regions, predominantly associated with light response, abiotic stress, hormone regulation, and growth and development, implying potential roles in various physiological processes. RNA-seq data showed distinct expression patterns of *StCLC* genes across different tissues, indicating tissue-specific expression. Furthermore, qRT-PCR results demonstrated that under NaCl treatment, the expression levels of all seven *StCLC* genes, including *StCLC3* and *StCLC6*, were significantly upregulated in roots, suggesting their active involvement in the response to salt stress.

**Discussion:**

These findings elucidate the structural, evolutionary, and functional diversity of the *StCLC* gene family and highlight its important role in salt stress response, thereby providing a theoretical foundation for the genetic improvement of salt tolerance in potato.

## 1 Introduction

Anion channels have been extensively studied in various tissues, cell types, and membranes of algae and higher plants, where they play central roles in cell signaling, osmotic regulation, plant nutrition, and metabolism (Barbier-Brygoo et al., 2000). In plant cells, these channels and transporters are essential for nutrient absorption, ion balance, and resistance to biotic or abiotic stress (Nguyen et al., 2016). Among them, chloride channel proteins (*CLC*) represent an important class of anion transporters distributed across organelles and plasma membranes, where they regulate key metabolic and physiological processes (Zifarelli and Pusch, 2010). The first *CLC* protein, *CLC-0*, was discovered in Torpedo marmorata in 1990 (Jentsch et al., 1990), and subsequent studies have revealed that *CLC* family genes are widely present in plants (Zifarelli and Pusch, 2010). Their physiological functions are broadly divided into two types: channel-type proteins that mediate passive Cl^-^ transport, and exchanger-type proteins that couple Cl^-^ transport with proton gradients (Picollo and Pusch, 2005). *CLC* members have been identified in *Arabidopsis thaliana* (Lv et al., 2009), *Oryza sativa* (Diédhiou and Golldack, 2006), and *Nicotiana tabacum* (Zhang et al., 2018), and in these and other crops, *CLC* genes have been shown to participate in tolerance to salinity, drought, and heavy metals.

Potato (*Solanum tuberosum*) is the fourth most important food crop worldwide, after maize, wheat, and rice, and contributes significantly to global food security through its high nutritional value, particularly as a source of carbohydrates, fiber, vitamins, and potassium (Zhang et al., 2017). It is cultivated in diverse agro-ecological zones, ranging from temperate regions of Europe and North America to the highlands of South America, Africa, and Asia, highlighting its adaptability and global significance. However, potato is highly sensitive susceptible to environmental stresses, and its productivity and quality are substantially reduced under unfavorable conditions (Suttle, 2008). Among these, salt stress is one of the most serious abiotic constraints, especially in irrigated regions affected by soil salinization, which currently accounts for nearly 20% of irrigated land worldwide (FAO, 2024; Liu et al., 2024). Salt stress exerts multiple adverse effects on plants, including osmotic stress, ionic imbalance, and oxidative damage, ultimately reducing growth and yield. Chloride ions accumulate excessively under saline conditions, and their mismanagement can cause toxicity. Thus, *CLC* proteins are critical regulators of Cl^-^ homeostasis, enabling efflux, sequestration, and redistribution to maintain cellular function under stress.

Evidence from other crops highlights the central role of *CLC* genes in salinity tolerance. In soybean, *GmCLC1* enhances salt resistance by reducing Cl^-^ transport to shoots and sequestering it in roots (Wei et al., 2016). In cotton, members of the *GhCLC* gene family are differentially expressed under salt stress, with *GhCLC5* and *GhCLC16* implicated in Cl^-^ and NO_3_^-^ homeostasis (Liu et al., 2020). In pomegranate, *PgCLC* antiporters contribute to vacuolar Cl^-^ sequestration in leaves and restriction of Cl^-^ uptake in roots, helping sustain ion balance during salt stress (Wu and Li, 2019; Liu et al., 2020). Recent work in Arabidopsis further demonstrates that *AtCLCf* can be relocalized from the Golgi to the plasma membrane under salinity, facilitating Cl^-^ efflux from roots as an adaptive mechanism (Liu et al., 2023; Rajappa et al., 2024). These studies collectively illustrate that plant *CLC* proteins, whether functioning in sequestration, efflux, or balancing Cl^-^ and other anions, play indispensable roles in conferring salt tolerance.

Despite the global significance of the potato and the well-established importance of *CLC* genes in other plants, systematic studies of the *CLC* gene family in potato remain limited. Addressing this gap, we carried out a comprehensive bioinformatics analysis of the *StCLC* gene family. Our study identified *CLC* members in the potato genome and examined their physicochemical properties, transmembrane topology, and predicted subcellular localization. Phylogenetic analyses were conducted to assess evolutionary relationships with *CLCs* in other species, while motif composition, gene structures, and conserved domains were analyzed to infer functional diversity. Chromosomal distribution and synteny analysis revealed patterns of duplication and conservation. *cis*-acting regulatory elements were identified in promoter regions, and Gene Ontology enrichment provided insights into potential biological roles. Finally, transcriptomic and qRT-PCR analyses were used to characterize *StCLC* expression profiles across tissues and in response to salt stress. Together, these integrated approaches provide new theoretical insights into the *CLC* gene family in potato and lay a foundation for future efforts to enhance salt tolerance in this globally important crop.

## 2 Materials and methods

### 2.1 Identification and physicochemical analysis of *CLC* members in the potato genome

The potato genome annotation files, nucleotide sequences, and amino acid sequence files were downloaded from the Potato Genome Sequencing Consortium (PGSC) database (Hardigan et al., 2016) (http://spuddb.uga.edu/index.shtml). Protein sequences of members of the Arabidopsis *CLC* gene family (*AtCLC*) were downloaded from the Arabidopsis TAIR database (http://www.arabidopsis.org/). Using the protein characteristic domains of the *CLC* gene family (labeled as PF00654) identified in the Pfam database (http://pfam.sanger.ac.uk/) for the model plant Arabidopsis *CLC* proteins, Using the specific structural domain (PF00654) of the *CLC* gene family, the HMMER (https://www.ebi.ac.uk/Tools/hmmer/) online software was used to search the potato genome sequences containing the *CLC* structural domain feature (E-value ≤ 1e^-5^). After removing redundant sequences, the obtained sequences were submitted to SMART (http://smart.embl-heidelberg.de) and NCBI CDD (https://www.ncbi.nlm.nih.gov/cdd) for screening, and sequences not containing the *CLC* structural domain were removed, ultimately identifying the members of the *StCLC* gene family. The online program ExPasy (Artimo et al., 2012) (https://web.expasy.org/protparam) was used to analyze the amino acid count, molecular weight, theoretical pI, and hydrophilicity of potato *CLC* family members. Transmembrane structure prediction was performed using the online program TMHMM 2.0 (https://services.healthtech.dtu.dk/services/TMHMM-2.0/). Subcellular localization prediction was performed using the online program Plant-mPLoc (Chou and Shen, 2010) (http://www.csbio.sjtu.edu.cn/bioinf/plant-multi/).

### 2.2 Protein tertiary structure and systematic tree analysis

To further investigate the functional and structural characteristics of *StCLC* proteins, the amino acid sequence of *StCLC* was input into SWISS-MODEL (Waterhouse et al., 2018) (https://swissmodel.expasy.org). Subsequently, the “Build Model” option was selected, and the resulting structure was exported as an image, thereby achieving the visualization of its three-dimensional structure. Protein sequences of *AtCLC* family members were downloaded from the Arabidopsis TAIR database, while sequences of tomato *SlCLC* family members were retrieved from the Ensembl Plants database (https://plants.ensembl.org/index.html). Use MEGA v11 software (website: https://www.megasoftware.net/) to perform multiple sequence alignment with the ClustalW algorithm under default parameters and find the most suitable model (Tamura et al., 2021). Construct a phylogenetic tree using the Maximum Likelihood (ML) method with the following parameter settings: 1000 replicates for the bootstrap method, select the Jones - Taylor - Thornton (JTT) model for Model/Method, choose Gamma Distributed (G) for RATES AND PATTERNS, and select Complete deletion for Gaps/Missing Data Treatment. Keep other parameters as default, and export the Newick file (Saitou, 1988). The phylogenetic tree was beautified and visualized using tvBOT (Xie et al., 2023) (https://www.chiplot.online/tvbot.html).

### 2.3 Protein motifs, conserved domain, and gene structures of *StCLCs*

The conserved motifs of the *StCLC* gene family were analyzed using the online protein motif analysis tool MEME (http://meme.nbcr.net/meme/cgi-bin/meme.cgi). The amino acid sequences of the StCLCs genes were input, with the parameters set as follows: the optimal matching length of conserved motifs was 6–100 amino acids (aa), the number of conserved motifs was set to 10, and all other parameters were kept at their default values (Nystrom and McKay, 2021). For subsequent visualization analysis, the mast.xml file was selected from the output results. The gene structure diagram was drawn using the online program GSDS (Hu et al., 2015) (https://gsds.cbi.pku.edu.cn/index.php). The conserved domains of *StCLCs* were analyzed using the Conserved Domain Database (Marchler-Bauer et al., 2015) (CDD, https://www.ncbi.nlm.nih.gov/cdd). Finally, TBtools v2.357 was used for visualization analysis.

### 2.4 Chromosome distribution and colinearity analysis

Based on the information obtained about the *StCLC* gene family members, chromosome localization information was retrieved from the Ensembl Plants database. The online program MG2C-2.1 (Chao et al., 2021) (http://mg2c.iask.in/mg2c_v2.1/) was used to generate chromosome localization maps. Synteny analysis was conducted on the *CLC* genes in potato (*Solanum tuberosum*), tomato (*Solanum lycopersicum*), Arabidopsis (*Arabidopsis thaliana*), rice (*Oryza sativa*), and pea (*Pisum sativum*) to determine the syntenic relationships among them. Visualization was achieved using the MCScanX algorithm and the Dual Synteny Plot program of TBtools v2.357 with default parameters (Chen et al., 2023).

### 2.5 Promoter *cis*-acting element analysis and GO annotation analysis

To reveal the regulatory characteristics of the *StCLC* promoter, the promoter sequence upstream of the *StCLC* start codon (2000 kb) was downloaded from NCBI. Trans-acting element analysis was performed using PlantCARE (Lescot, 2002) (http://bioinformatics.psb.ugent.be/webtools/plantcare/html/) The results of the *cis*-acting regulatory element analysis were visualized using TBtools v2.357.Perform GO (Gene Ontology) enrichment analysis of *StCLC* protein sequences using the online program DAVID (Sherman et al., 2022) (https://david.ncifcrf.gov/home.jsp).

### 2.6 *In-silico* expression of *StCLCs* in different tissues

Download potato transcriptome data from the Potato Genome Sequencing Consortium (PGSC) database. Retrieve the gene IDs of *StCLC* from the database and retrieve their FPKM (Fragments Per Kilobase of exon model per Million mapped fragments) values in different tissues (Mortazavi et al., 2008) (Leaves, Roots, Flowers, Petals, Buds, Sepals, Stolons, and Petioles). To eliminate technical biases between samples, the FPKM values were normalized using the TMM (Trimmed Mean of M-values) method implemented in the edgeR software package. The FPKM values were log_10_-transformed. Differentially expressed genes (DEGs) were identified using DESeq2 with a false discovery rate (FDR) adjusted p-value < 0.05. Genes with an average FPKM > 0 across all tissues were considered expressed. The expression heatmap was generated using TBtools v2.357 software.

### 2.7 Plant materials and treatments

Potato ‘Desiree’ seedlings were grown hydroponically under conditions of 25°C/20°C (day/night), relative humidity of 65%-75%, and a photoperiod of 16h/8h (day/night), using Hoagland nutrient solution for irrigation (Gong et al., 2023). The seedlings were irrigated with Hoagland nutrient solution every 2 days. After 3 weeks, they were treated with 0 (control) and 200 mmol/L NaCl solutions. Leaves and roots were collected from the plants at 0, 3, 6, 12, 24, and 48 hours after salt treatment (three biological replicates were performed at each time point to ensure statistical validity), rapidly frozen in liquid nitrogen, and stored at -80°C until further analysis.

### 2.8 RNA extraction, cDNA synthesis, and qRT-PCR

Total RNA was extracted from potato leaves and roots using Trizol reagent (Sangon Biotech, China) according to the manufacturer’s protocol. After extraction, the first strand of cDNA was synthesized using PrimeScript™RT Master Mix (Perfect Real Time) (Takara, Japan) according to the manufacturer’s protocol. Specific primers were designed using Primer Premier 5 (Singh et al., 1998) and synthesized by Sangon Biotech (Shanghai, China). ef1α (LOC102577640) was used as the housekeeping gene. Amplification was performed on the BIOER QuantGene 9600 Fluorescent Quantitative PCR Instrument, using the TB Green^®^ Premix Ex Taq™ II Reagent Kit (Tli RNaseH Plus) (Takara, Dalian, China). Each treatment group included 3 biological replicates (independent samples taken from different plants), with each biological sample having 3 technical replicates (parallel reactions using the same cDNA template). The qRT-PCR procedure is as follows: (і) predenaturation at 95°C for 2min, (ii) 40 cycles of amplification: denaturation at 95°C for 5s, annealing at 55°C for 30s, extension at 72°C for 1min, and (iii) extension at 72°C for 10min. The expression levels of genes were analyzed using the 2−^ΔΔCT^ method. Significance was analyzed using SPSS, and relative gene expression levels were visualized using GraphPad Prism 9.

## 3 Results

### 3.1 Identification of *CLC* gene family members in potato

Seven *CLC* genes were identified from the potato genome, and they were named *StCLC1* to *StCLC7* based on their chromosomal locations. The physicochemical properties of *StCLC* proteins (**Table 1**) indicate that the amino acid count encoded by *StCLC* family members ranges from 752 to 784 AA, with molecular weights between 80.21 and 86.45 kDa. The theoretical isoelectric point (pI) ranges from 6.18 to 8.84, and the total average hydrophilicity coefficient ranges from 0.053 to 0.394, indicating that all seven *StCLC* proteins are hydrophobic. They have 8 to 12 transmembrane domains, and subcellular localization analysis shows that all seven *StCLC* proteins are expressed on the cell membrane. Additionally, *StCLC5* and *StCLC6* are also expressed on the mitochondrial membrane, which suggests that *StCLC* may be a transmembrane protein.

**Table 1:** The gene and protein characteristics of the *StCLC* gene family in *Solanum tuberosum*.

Gene name	Transcript ID	AA	MW(kDa)	pI	GRAVY	TMHs	Subcellular localization
*StCLC1*	PGSC0003DMT400064087	775	84.32	8.55	0.363	8	Cell membrane
*StCLC2*	PGSC0003DMT400017962	784	86.45	8.76	0.312	8	Cell membrane
*StCLC3*	PGSC0003DMT400052072	774	86.10	6.8	0.341	10	Cell membrane
*StCLC4*	PGSC0003DMT400078919	767	84.38	8.84	0.389	12	Cell membrane
*StCLC5*	PGSC0003DMT400057279	752	80.21	6.18	0.053	8	Cell membrane Mitochondrion
*StCLC6*	PGSC0003DMT400029204	756	80.81	6.23	0.089	10	Cell membrane Mitochondrion
*StCLC7*	PGSC0003DMT400025239	776	84.73	8.5	0.394	10	Cell membrane

AA, amino acid number; MW, molecular weight; pI, isoelectric point; GRAVY, grand average of hydro-pathicity; TMHs, number of predicted TMHs.

### 3.2 Tertiary structure of *StCLC* proteins and phylogenetic analysis of the gene family

**Figure 1:**
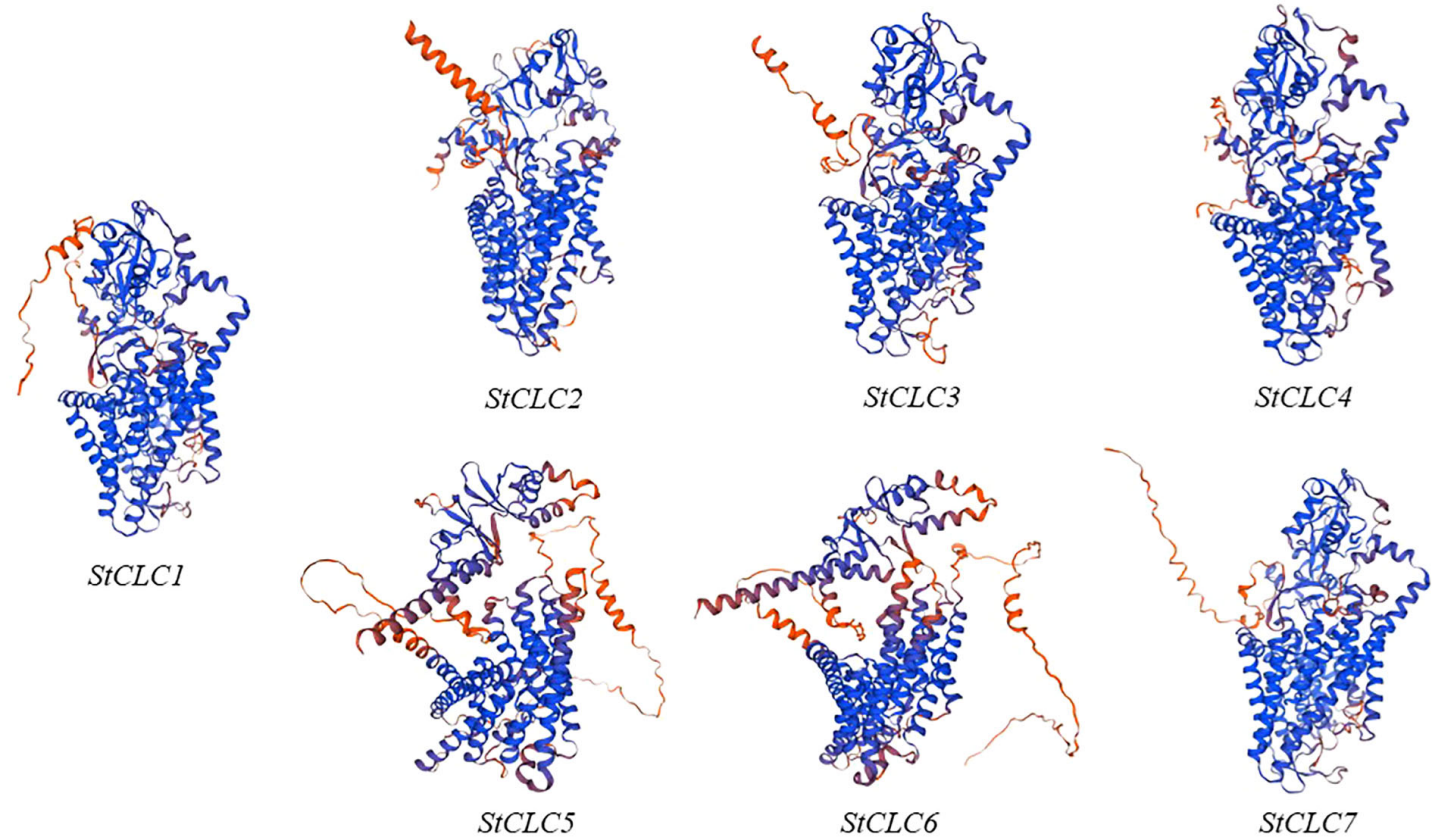
The three-dimensional structures of seven genes in the *StCLCs* family predicted using Swiss-Model.

**Figure 2:**
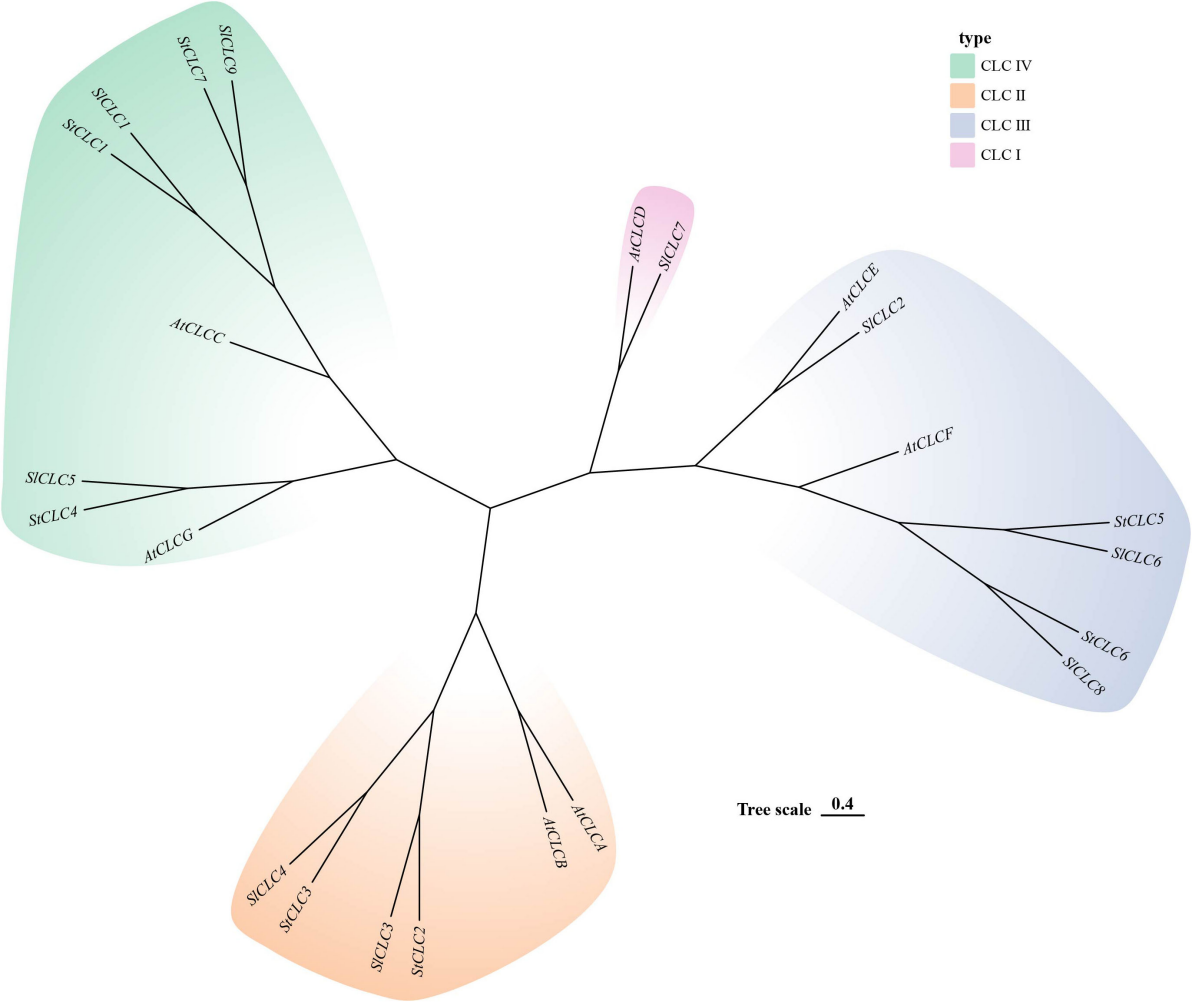
Phylogenetic analysis of potato (*Solanum tuberosum*, *StCLC*), Arabidopsis (*Arabidopsis thaliana*, *AtCLC*),and tomato (*Solanum lycopersicum*, *SlCLC*). Different colored regions represent four subfamilies: CLC IV (light green), CLC II (light orange), CLC III (light blue), and CLC I (pink). The “Tree scale 0.4” indicates the evolutionary distance.

The prediction of the three-dimensional structures of *StCLC* proteins revealed distinct structural characteristics among *StCLC* proteins (**Figure 1**). *StCLC1*, *StCLC2*, *StCLC3*, *StCLC4*, and *StCLC7* exhibited relatively compact and well-organized 3D architectures, with prominent helical domains (displayed in blue) and some disordered or flexible regions (in orange). In contrast, *StCLC5* and *StCLC6* showed more dispersed structural patterns. These structural variations among *StCLC* proteins may imply differences in their spatial folding patterns, which could further relate to their functional diversities in biological processes. To clarify the evolutionary relationships within the *StCLC* gene family, members of the *CLC* gene families from potato, tomato, and Arabidopsis were selected in this study to construct a phylogenetic tree (**Figure 2**). Based on the classification criteria of Arabidopsis and tomato *CLC* genes, the *StCLC* family was divided into 3 clusters: Cluster CLCII contains 2 *StCLC* family members, namely *StCLC2* and *StCLC3*; Cluster CLCIII includes 2 *StCLC* family members, specifically *StCLC5* and *StCLC6*; and Cluster CLCIV comprises 3 *StCLC* family members, which are *StCLC1*, *StCLC4*, and *StCLC7*.

### 3.3 Conserved motifs, conserved domains and gene structure analysis of the *StCLCs*

**Figure 3:**
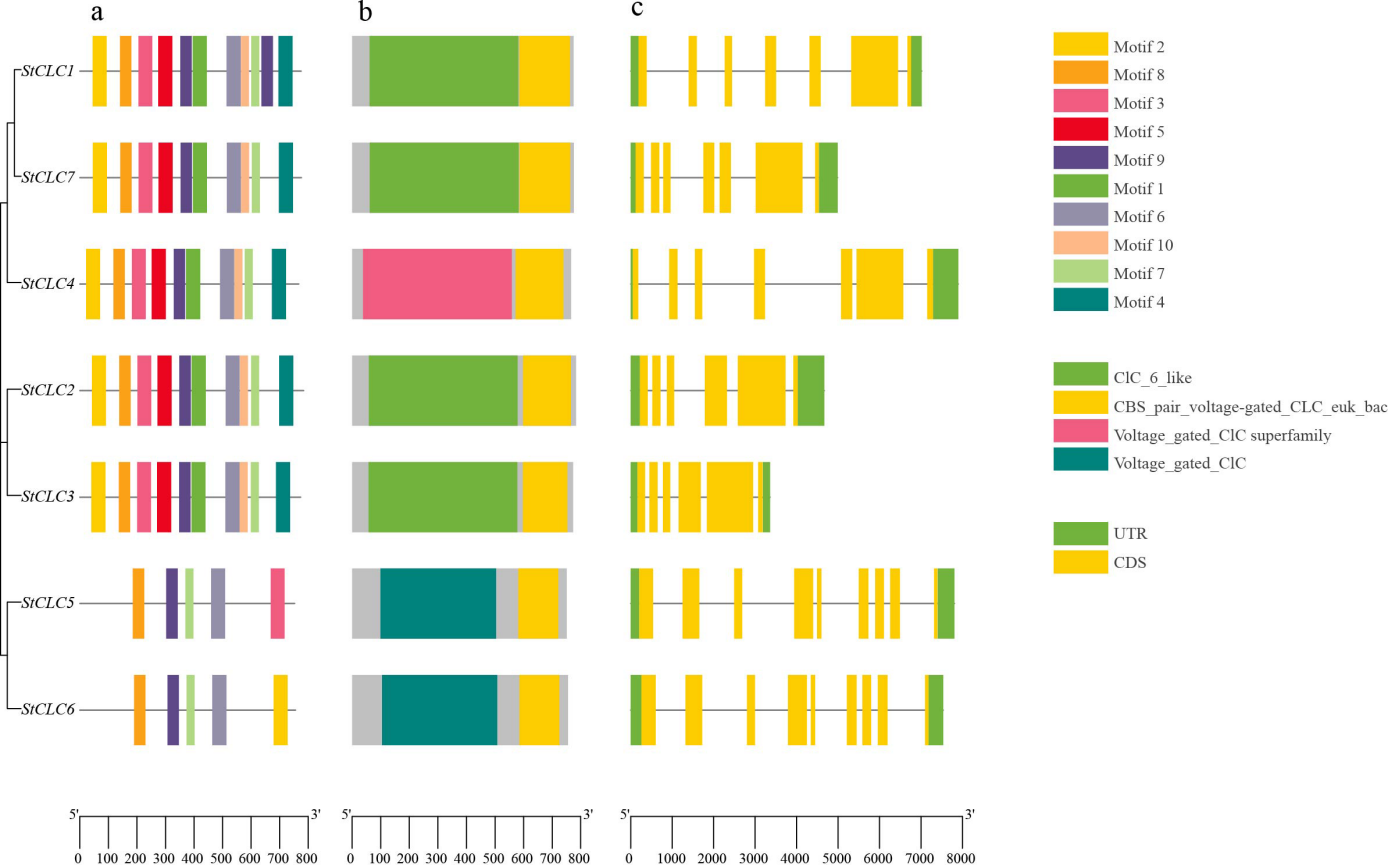
Conserved motifs, conserved domains, and gene structure analysis of the *StCLCs* gene family. **(a)** Schematic diagram of the amino acid motif of *StCLCs*. Different colors of the boxes represent different motifs. **(b)** Schematic representation of the conserved domains of *StCLCs*. Different colored boxes represent different conserved domains. **(c)** Illustration of the CDS/UTR of the corresponding *StCLC* genes. The yellow boxes indicate the CDS, while the green boxes indicate the UTR.

To further analyze the structure and function of *StCLC* members, we conducted conserved Motifs, conserved domain, and gene structure analyses on seven *StCLC* members. The results of the conserved Motif analysis (**Figure 3a**) showed that all seven *StCLC* members contained Motif6, Motif7, Motif8, and Motif9, which were in the middle part of the *CLC* domain and constituted most of the *CLC* domain. *StCLC1*, *StCLC2*, *StCLC3*, *StCLC4*, and *StCLC7* all contain Motif1, 2, 3, 4, 5, 6, 7, 8, 9, and 10, which are respectively located at the N-terminal, C-terminal, or middle regions of the *CLC* domain. In addition to Motif6, Motif7, Motif8, and Motif9, *StCLC5* also contains Motif3, and *StCLC6* also contain Motif2. The results of the conserved domain analysis (**Figure 3b**) indicate that all *StCLC* proteins contain the CBS_pair_voltage-gated_CLC_euk_bac domain. Additionally, *StCLC1*, *StCLC2*, *StCLC3*, and *StCLC7* all contain the CLC_6_like domain, *StCLC4* contains the Voltage_gated_CLC superfamily domain, and *StCLC5* and *StCLC6* both contain the Voltage_gated_CLC domain. Gene structure analysis indicates (**Figure 3c**) that there is no significant difference in the number of exons and introns among *StCLC* genes. *StCLC1*, *StCLC4*, and *StCLC7* contain 7 exons and 6 introns, while *StCLC2* and *StCLC3* contain 6 exons and 5 introns. *StCLC5* and *StCLC6* contain 9 exons and 8 introns.

### 3.4 Chromosomal distribution and synteny analysis of the *StCLCs*

**Figure 4:**
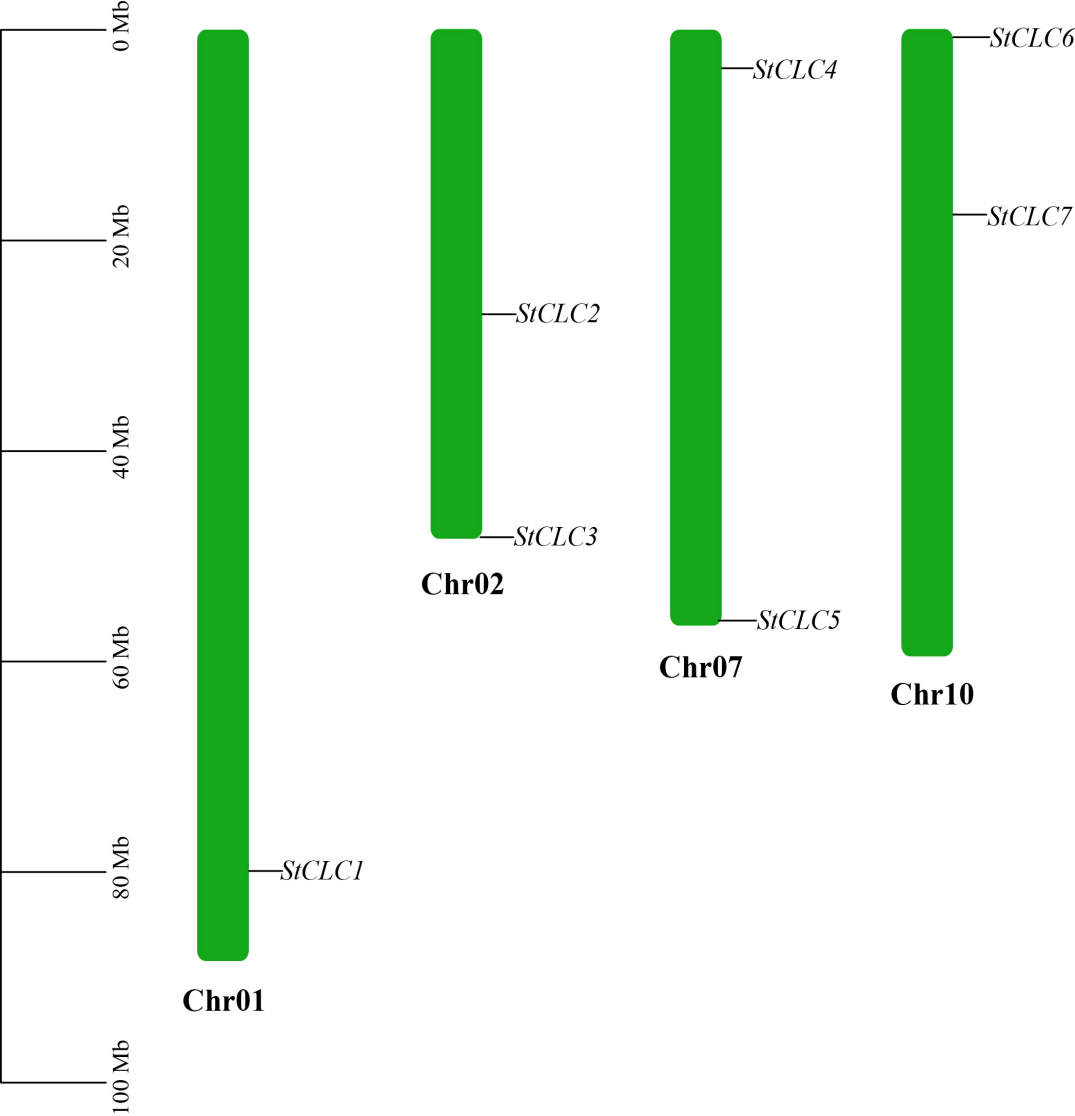
Chromosomal distribution of the *StCLC* gene family. The bars represented chromosomes, and chromosome numbers were indicated at the right of each bar with length marked at the left of whole figure.

**Figure 5:**
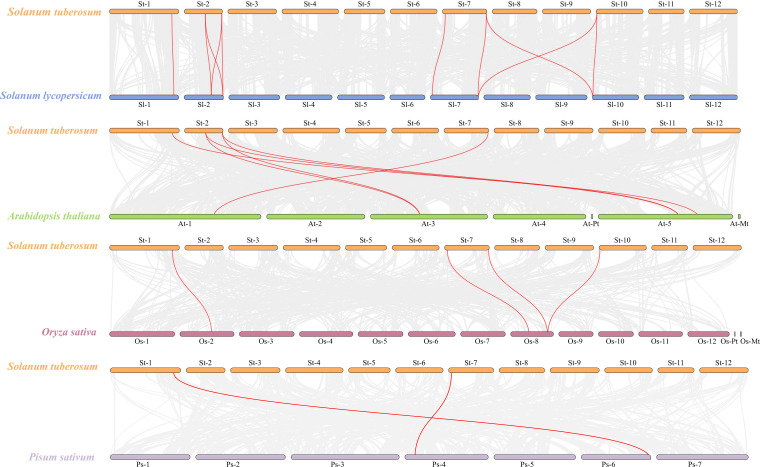
Synteny analysis of *CLC* genes between potato (*Solanum tuberosum*, St) and tomato (*Solanum lycopersicum*, Sl), Arabidopsis (*Arabidopsis thaliana*, At), rice (*Oryza sativa*, Os), and pea (*Pisum sativum*, Ps). The gray lines in the background represent collinear blocks in the genomes of potato, tomato, Arabidopsis, rice, and pea, while the red lines highlight the homologous pairs of *CLC* genes.

**Figure 6:**
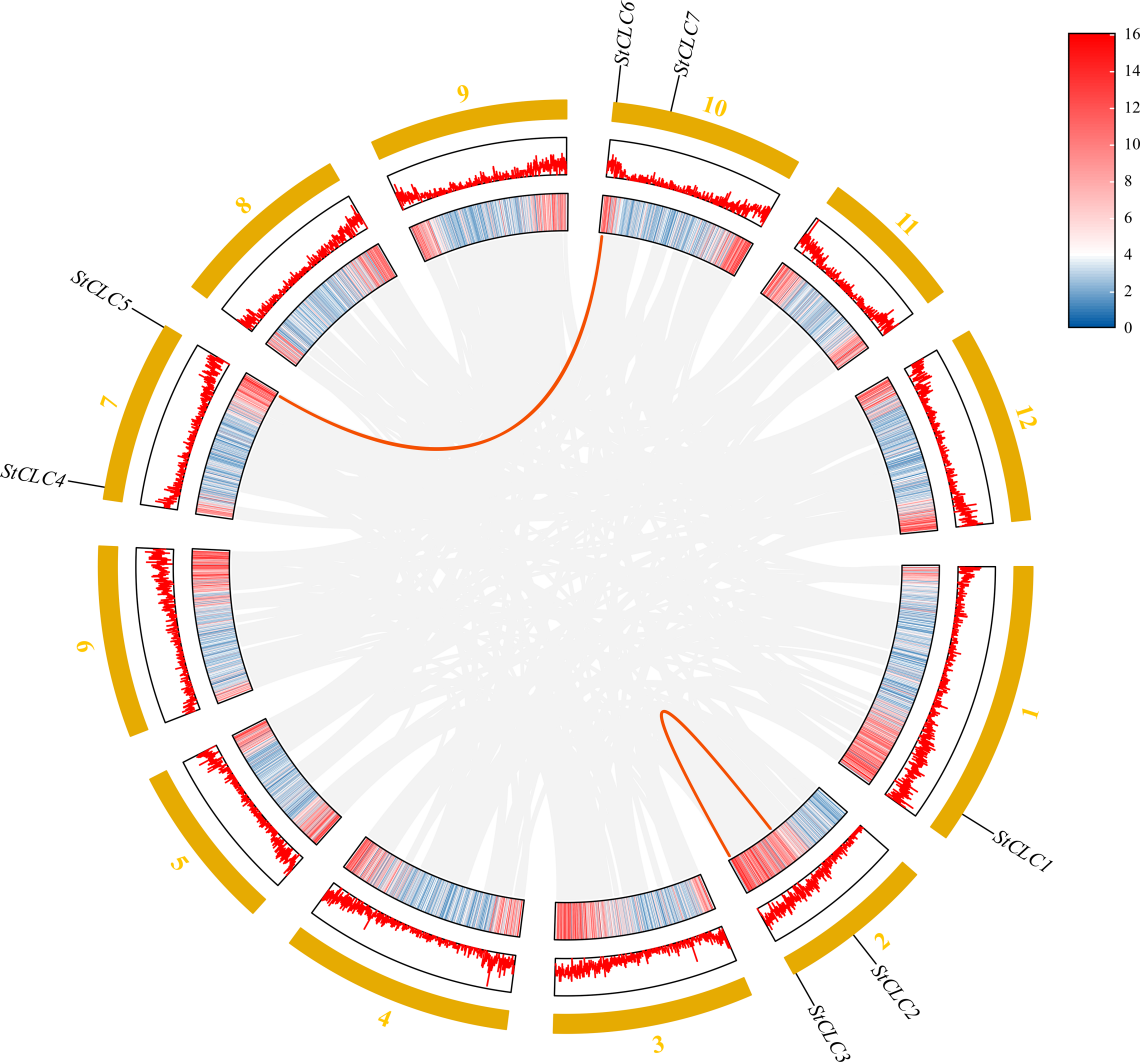
Homology analysis of *StCLC* genes in potatoes. Gray lines indicate all homologous blocks in the genome of potato, and the red lines indicate duplicated *StCLC* gene pairs.

Members of the *StCLC* gene family are distributed across four chromosomes: Chr1, Chr2, Chr7, and Chr10 (**Figure 4**). There is one gene on Chr1, and two genes each on Chr2, Chr7, and Chr10. No genes are present on chromosomes Chr3, Chr4, Chr5, Chr6, Chr8, Chr9, Chr11, or Chr12.To further infer the evolutionary relationships among *CLC* genes in plants, we conducted intrachromosomal synteny analysis was performed on the *CLC* genes in potato (*Solanaceae*), tomato (*Solanaceae*), Arabidopsis thaliana (*Brassicaceae*), rice (*Poaceae*), and pea (*Fabaceae*). The results showed that there were 10 pairs of collinear *CLC* genes between potato and tomato, 6 pairs of collinear *CLC* genes between potato and Arabidopsis thaliana, 4 pairs of collinear *CLC* genes between potato and rice, and 2 pairs of collinear *CLC* genes between potato and pea (**Figure 5**). These findings indicate that the *CLC* genes of potato have a closer evolutionary relationship with those of tomato, suggesting that the two may share a common ancestor. Intraspecific synteny analysis of potato *CLC* genes revealed that the *StCLC2* and *StCLC3* genes exhibited synteny on Chr2; in contrast, the *StCLC5* and *StCLC6* genes showed synteny on Chr7 and Chr10, respectively (**Figure 6**). This result implies that the expansion in the number of members of the *StCLC* gene family may be attributed to gene duplication events.

### 3.5 Gene ontology enrichment analysis of *StCLCs*

**Figure 7:**
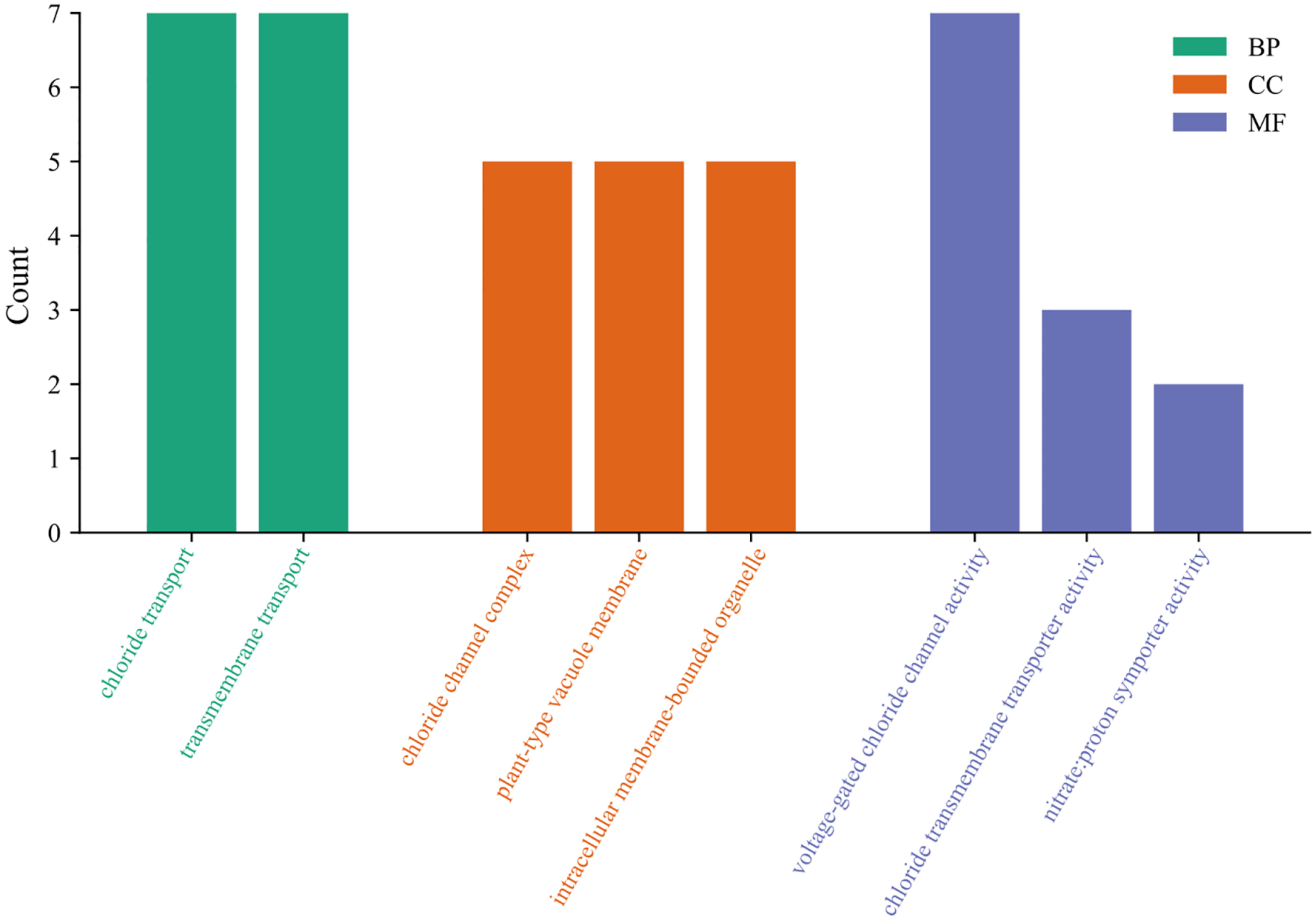
GO analysis of *StCLC* genes. The vertical axis represents the number of genes involved in the cor-responding classification, and the horizontal axis represents the functional categories of *StCLC* genes. Green rectangles represent biological processes (BP), orange rectangles represent cellular components (CC), and purple rectangles represent molecular functions (MF).

To reveal the functional classification of *StCLC* genes, the GO enrichment pathways of *StCLC* were analyzed from three aspects: biological process (BP), cellular component (CC), and molecular function (MF) (**Figure 7**). In biological processes, most *StCLC* genes play a role in chloride transport and transmembrane transport. In terms of molecular function, it is associated with chloride channel complex and plant-type vacuole membrane. Among the cell components, all members are related to the activity of voltage-gated chloride channels. In addition, some members are related to the activity of chloride transmembrane transporters and the activity of nitrate proton symporters. The results indicate that the *StCLC* gene plays many important roles in potatoes, the most significant of which is its involvement in chloride ion transport.

### 3.6 Analysis of promoter *cis*-acting elements in the *StCLC* gene family

**Figure 8:**
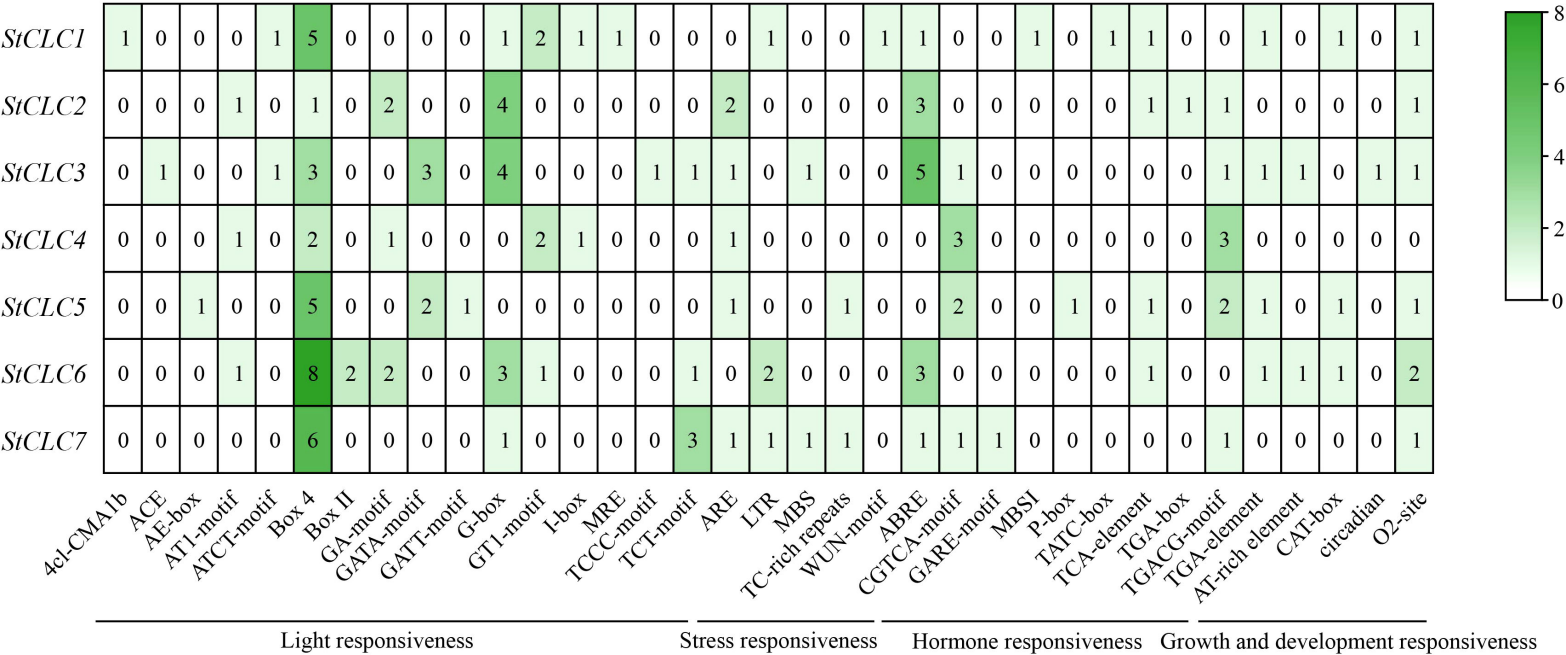
Analysis of *cis*-acting regulatory elements upstream of the *StCLCs* promoter within 2000 bp. The rows represent different *StCLC* genes (from *StCLC1* to *StCLC7*), and the columns represent various cis - acting elements. These elements are categorized into functional groups such as light responsiveness, stress responsiveness, hormone responsiveness, and growth and development responsiveness. The color gradient (from white to dark green) and the numerical values in the cells indicate the frequency or abundance of each *cis*-acting element in the promoter region of the corresponding *StCLC* gene.

To explore the *cis*-acting regulatory elements of *StCLCs*, the sequence upstream of the translation start site of *StCLCs* within 2000 bp was analyzed using the online tool PlantCARE. In this study, 35 distinct *cis*-acting regulatory elements were identified in the promoter region of *StCLCs* (**Figure 8**). All *cis*-acting regulatory elements can be broadly categorized into four major classes: light responsive elements, stress responsive elements, hormone responsive elements, and plant growth and development responsive elements. Among these, *StCLCs* primarily contain light responsive elements Box4, G-box, and abscisic acid response elements (ABRE). Additionally, they contain methyl jasmonate response elements (CGTCA-motif, TGACG-motif), light responsive elements (GA-motif, GATA-motif), corn solubilin protein metabolic regulation response elements (O2-site), antioxidant response elements (ARE), low temperature response elements (LTR), drought induced response elements (MBC), and defense and stress response elements (TC-rich repeats) et al., These results suggest that the expression of *StCLCs* genes may be regulated by light, environmental, and hormonal signals, among others, and that *StCLCs* may play an important role in potato growth and development under abiotic stress conditions.

### 3.7 Analysis of *StCLC* gene expression in different tissues

**Figure 9:**
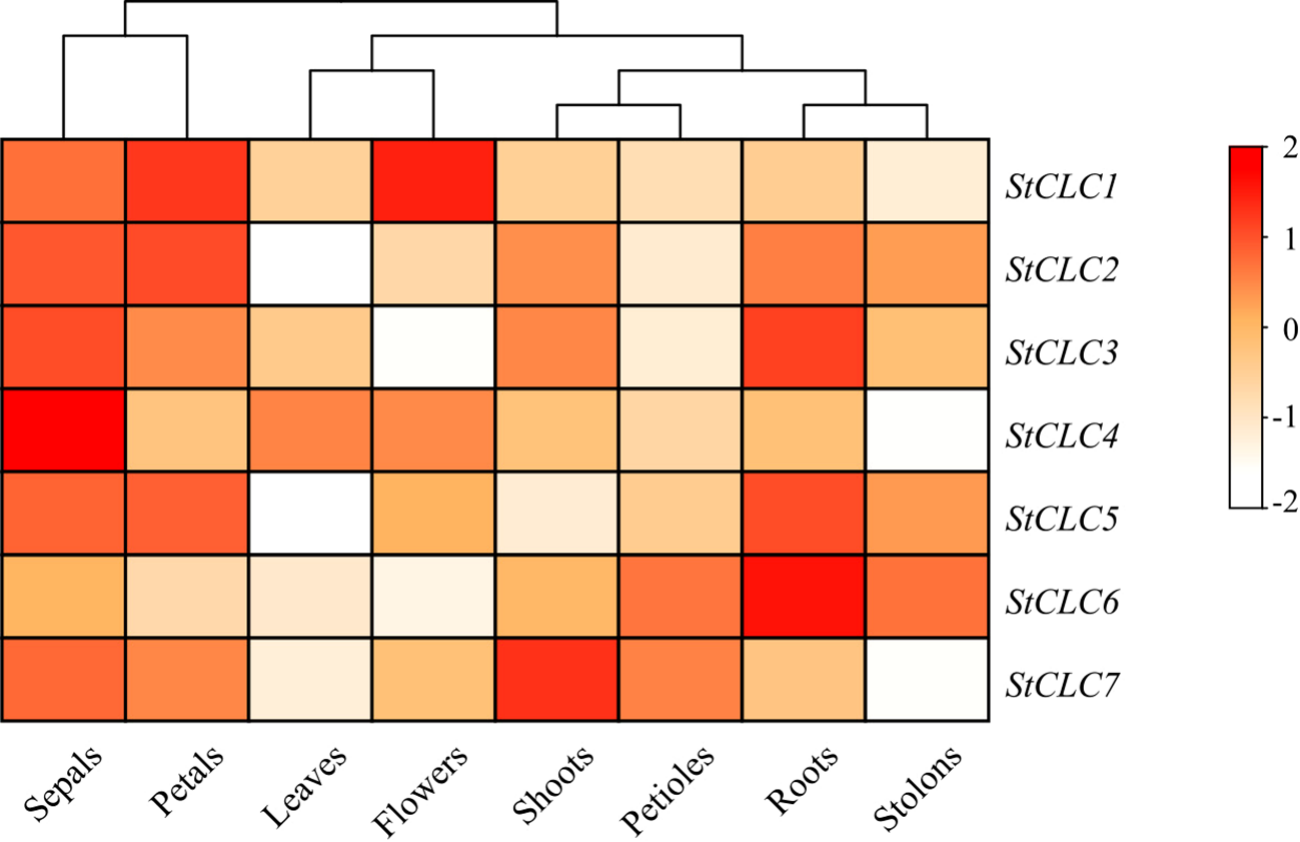
Expression analysis of potato *StCLC* in eight tissues. The heatmap presents the relative expression levels of genes through a color gradient (from white to dark red). The color scale on the right shows that the redder the color, the higher the gene expression level (the value range is from -2 to 2; the larger the positive value and the darker the red color, the higher the expression level; negative values or lighter colors indicate lower expression levels). The relative expression levels of *StCLC* genes were normalized using Log_10_.

To elucidate the potential function of the *StCLC* gene in potato growth and development, RNA-Seq data for the *StCLC* gene in different tissues (leaves, roots, flowers, petals, buds, sepals, stolons, and petioles) were downloaded from the potato transcriptome database. The analysis results showed that the *StCLC* gene is expressed in most tissues, with higher expression levels in sepals, petals, and roots (**Figure 9**). *StCLC1* is highly expressed in flowers and petals, *StCLC2* is highly expressed in petals, *StCLC3*, *StCLC5*, and *StCLC6* are highly expressed in roots (with *StCLC6* showing the highest expression in roots), *StCLC4* is highly expressed in sepals, and *StCLC7* is highly expressed in buds.

### 3.8 Expression pattern of *StCLC* genes under salt stress

**Figure 10:**
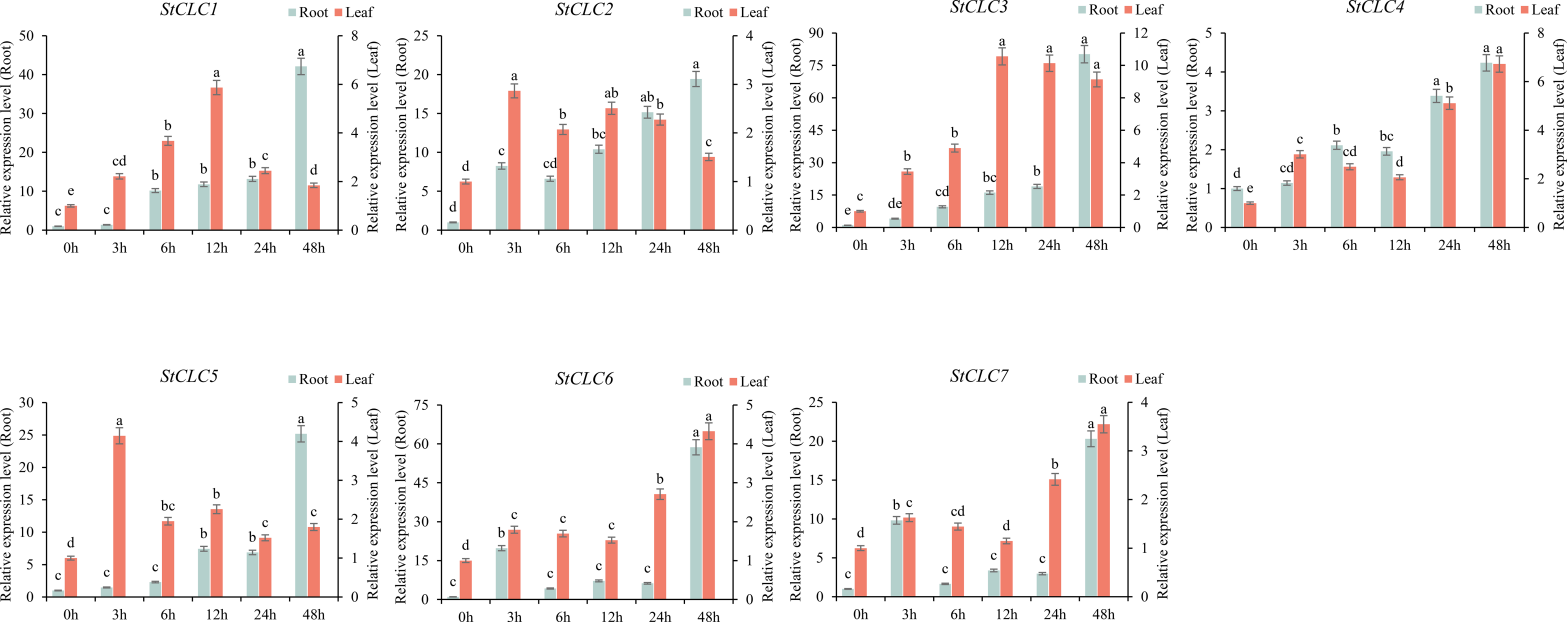
Relative expression levels of the *StCLC* gene in roots and leaves under 200 mmol/L NaCl treatment. Data are expressed as mean ± standard error of the mean (SEM). Bar charts with different letters within the same group indicate significant differences between different treatment times (p < 0.05).

To analyze the response of *StCLCs* genes to salt stress treatment, qRT-PCR was used to analyze the gene expression of *StCLCs* in potato material ‘Desiree’ under NaCl treatment at 0, 3, 6, 12, 24, and 48 hours. The results showed (**Figure 10**) that in roots, the expression levels of *StCLC1*, *StCLC2*, *StCLC3*, *StCLC4*, and *StCLC5* exhibited the same trend, increasing with increasing stress duration and reaching a peak at 48 h. *StCLC6* and *StCLC7* showed a significant increase in expression levels from 0 to 3 hours of salt stress treatment, a significant decrease from 3 to 6 hours, stable expression levels from 6 to 24 hours, a significant increase from 24 to 48 hours, and peak expression levels at 48 hours. In leaves, the expression levels of *StCLC1*, *StCLC2*, and *StCLC5* showed the same trend, increasing initially and then decreasing with increasing salt stress duration. Among these, *StCLC1* reached its peak at 12 hours, *StCLC2* and *StCLC5* reached their peaks at 3 hours, and *StCLC3*, *StCLC6* showed an overall increase in expression levels with increasing salt stress duration, with *StCLC3* reaching its peak at 12 hours and *StCLC6* at 48 hours. *StCLC4* and *StCLC7* exhibited the same trend, with expression levels significantly increasing from 0 to 3 hours, decreasing from 3 to 12 hours, and then significantly increasing again from 12 to 48 hours, reaching their peak at 48 hours. These results indicate that *StCLC* genes are significantly induced by salt stress. Like other crop *CLC* gene family members, *StCLCs* may actively respond to salt stress and participate in the process of resisting salt stress.

## 4 Discussions

The identification of seven *StCLC* genes in the potato genome aligns with the number of *CLC* genes reported in Arabidopsis (7 *AtCLC* genes; Hechenberger et al., 1996) but is fewer than in tomato (9 *SlCLC* genes; Ma et al., 2025), rice (10 *OsCLC* genes; Diédhiou and Golldack, 2006), and tobacco (17 *NtCLC* genes; Zhang et al., 2018). This variation in gene family size likely reflects differences in genome size, ploidy levels, and gene duplication events across species. For instance, potato, a tetraploid species, has undergone extensive segmental and tandem duplications, which may have shaped the *StCLC* gene family size (Wang et al., 2018). The physicochemical properties of *StCLC* proteins (e.g., amino acid counts of 752–784, molecular weights of 80.21–86.45 kDa, and hydrophobic nature) are consistent with those of *CLC* proteins in other plants, indicating conserved structural roles as transmembrane anion transporters (Zifarelli and Pusch, 2010). The presence of 8–12 transmembrane domains and their localization primarily to the cell membrane, with *StCLC5* and *StCLC6* also localized to the mitochondrial membrane, suggests functional specialization. Mitochondrial localization may indicate roles in regulating anion fluxes in energy-related processes, as observed in Arabidopsis *AtCLCd* and *AtCLCf* (De Angeli et al., 2009).

Protein three - dimensional structure prediction shows that all *StCLC* proteins (*StCLC1*-*StCLC7*) in potato exhibit a similar overall folding pattern, which is consistent with the characteristics of the *CLC* protein family. As shown in the research on *CLC* protein structure in relevant literature (Dutzler et al., 2002), this conserved folding mode indicates that they retain the core functional domains responsible for anion transport. The presence of multiple α - helical structures (represented by the coiled blue parts in the figure1) is consistent with the typical transmembrane domain architecture of *CLC* proteins. In plants, like the *CLC* proteins in Arabidopsis (Yang et al., 2023), the transmembrane structure is the key to mediating the flow of anions across cell membranes, which is crucial for maintaining cellular ion homeostasis. In potato, this transmembrane structure of *StCLC* proteins is also very likely to play a similar role in the transmembrane transport of anions such as chloride ions.

During the long - term evolution of potato, gene loss events may have occurred, resulting in the absence of Cluster I genes. According to studies on plant gene family evolution, gene loss is usually related to the adaptation of species to specific environments. As a tuber-bearing crop, potato has unique physiological characteristics. Compared with Arabidopsis, it may have developed alternative mechanisms for nitrate storage and utilization. For example, Arabidopsis relies on *AtCLCa* in Cluster I to regulate nitrate accumulation in vacuoles (De Angeli et al., 2006). In potato, other genes or pathways in the *CLC* gene family or other gene families may undertake the function of nitrate regulation. This makes Cluster I genes no longer necessary for the survival and development of potato, so they are gradually lost during evolution (Helsen et al., 2020). Although the potato genome has been sequenced, there may still be problems of incomplete genome assembly and annotation. Current genome assembly algorithms may have difficulties in accurately assembling repetitive regions or regions with complex structures. If Cluster I genes in potato are located in these regions, they may not be accurately identified or annotated. Some studies have shown that genes with low expression levels or specific expression patterns are more likely to be missed during the annotation process (Li et al., 2022). Clustering analysis of *StCLC* genes with *CLC* genes from tomato (Solanaceae), Arabidopsis thaliana (Brassicaceae), rice (Poaceae), and pea (Fabaceae) showed that the homology between *StCLC* genes and tomato CLC genes (with 10 collinear gene pairs) was higher than that between *StCLC* genes and Arabidopsis thaliana *CLC* genes (6 collinear gene pairs), rice *CLC* genes (4 collinear gene pairs), and pea *CLC* genes (2 collinear gene pairs) (**Figure 5**). There exists a conserved evolutionary relationship of *CLC* genes within *Solanaceae* plants. Compared with *CLC* genes from non-*Solanaceae* families (*Brassicaceae*, *Poaceae*, *Fabaceae*), *StCLC* genes (from potato, a *Solanaceae* plant) show a closer clustering tendency with tomato *CLC* genes (also from *Solanaceae*). This clustering pattern is consistent with the taxonomic and phylogenetic hierarchy of plants, reflecting that *CLC* genes in plants of the same family have retained more ancestral sequence characteristics and evolutionary consistency during long-term evolution (Spooner et al., 2025; Ma et al., 2025) The syntenic relationship between potato *StCLC* genes and *CLC* genes from *Arabidopsis thaliana* (At, *Brassicaceae*), *Oryza sativa* (Os, *Poaceae*), and *Pisum sativum* (Ps, *Fabaceae*) is significantly sparser. Among these, the number of collinear gene pairs between *StCLC* genes and Arabidopsis thaliana *CLC* genes is greater than that between *StCLC* genes and rice or pea *CLC* genes, while the number of collinear gene pairs with rice and pea is even smaller (as indicated by the relatively sparse red connecting lines between St and At, Os, Ps in the figure). This phenomenon demonstrates that the taxonomic family difference among plants increases—especially when crossing from dicotyledonous plants (*Arabidopsis thaliana*) to monocotyledonous plants (rice) and across different dicotyledonous families (pea)—the synteny of *CLC* genes decreases significantly. It further verifies that for the clustering distance and synteny degree between *StCLC* genes and *CLC* genes from plants of different families, the more distant the phylogenetic *relationship* of the species, the weaker the gene synteny. Meanwhile, although the synteny between potato *CLC* genes and *CLC* genes from non-Solanaceae plants is relatively weak, there are still a certain number of collinear gene pairs (e.g., the red connecting lines between St and At, Os, and Ps in the figure). This also implies that the *CLC* gene family has an ancient origin, and these collinear genes may be derived from a common ancestral gene of early land plants, which provides a foundation for maintaining the conserved functions (such as chloride ion transport) of *CLC* genes across different plant groups (Wang et al., 2010). Intraspecific synteny analysis revealed tandem duplications (e.g., *StCLC2*/*StCLC3* on Chr2 and *StCLC5*/*StCLC6* on Chr7/Chr10; **Figure 6**), which likely contributed to the expansion of the *StCLC* gene family. Such duplications are common in plant genomes and often lead to functional redundancy or neofunctionalization (Panchy et al., 2016). For instance, *StCLC2* and *StCLC3*, being syntenic, may share similar roles in chloride transport, as tandemly duplicated genes often retain conserved functions (Cannon et al., 2004; Zhou et al., 2024).

The conserved motif and domain analyses (**Figures 3a, b**) revealed that all *StCLC* proteins possess the CBS_pair_voltage-gated_CLC_euk_bac domain, a hallmark of *CLC* proteins involved in anion selectivity and gating (Estévez and Jentsch, 2002). The presence of additional domains, such as CLC_6_like in *StCLC1*, *StCLC2*, *StCLC3*, and *StCLC7*, and Voltage_gated_CLC in *StCLC4*, *StCLC5*, and *StCLC6*, suggests functional diversification within the family. For example, the Voltage_gated_CLC domain is associated with voltage-dependent anion transport, which may be critical for ion homeostasis under stress conditions (Zifarelli and Pusch, 2010). The gene structure analysis (**Figure 3c**) showed variability in exon-intron organization, with *StCLC5* and *StCLC6* having more exons (9) than others (6–7). This structural divergence may reflect adaptations in gene regulation or alternative splicing, as observed in rice *OsCLC* genes (Diédhiou and Golldack, 2006). The conserved motifs (Motif6, Motif7, Motif8, Motif9) across all *StCLCs* indicate a core functional region within the *CLC* domain, likely responsible for anion transport, while additional motifs in specific *StCLCs* (e.g., Motif3 in *StCLC5*, Motif2 in *StCLC6*) suggest potential functional specialization.

The distribution of *StCLC* genes across four chromosomes (Chr1, Chr2, Chr7, Chr10) with no genes on other chromosomes (**Figure 4**) suggests a non-random genomic organization, possibly driven by evolutionary constraints or selection pressures. The clustering of *StCLC2*/*StCLC3* on Chr2 and *StCLC5*/*StCLC6* on Chr7/Chr10 supports the hypothesis of tandem and segmental duplications as mechanisms for gene family expansion. Such duplications are critical for generating genetic diversity and enabling plants to adapt to environmental stresses (Hanada et al., 2008). The synteny analysis (**Figures 5**, **6**) further corroborates that gene duplication events, particularly tandem duplications, have shaped the *StCLC* gene family, like patterns observed in tomato and Arabidopsis (Lv et al., 2009; Ma et al., 2025).

Gene ontology (GO) enrichment analysis (**Figure 7**) highlighted the primary roles of *StCLC* genes in chloride transport, transmembrane transport, and voltage-gated chloride channel activity. These functions are consistent with the roles of *CLC* genes in other plants, such as Arabidopsis *AtCLCc*, which regulates chloride homeostasis in roots under salt stress (Jossier et al., 2010). The association of *StCLCs* with nitrate proton symporter activity suggests a dual role in chloride and nitrate transport, as observed in rice *OsCLC1*, which facilitates nitrate accumulation under salt stress (Diédhiou and Golldack, 2006). This dual functionality could be critical for potato, an economically important crop, as nitrate uptake is essential for growth and tuber development (Marschner, 2012). The involvement of *StCLCs* in plant-type vacuole membranes further supports their role in vacuolar ion sequestration, a key mechanism for maintaining cellular ion balance under salt stress (Blumwald, 2000; Mansour, 2023).

The identification of 35 *cis*-acting regulatory elements in *StCLC* promoter regions (**Figure 8**) underscores their complex regulation under various environmental and hormonal signals. The prevalence of light-responsive elements (e.g., Box4, G-box) suggests that *StCLC* expression is modulated by light, which may influence diurnal ion transport cycles, as reported in Arabidopsis (Marmagne et al., 2007).Stress-responsive elements, such as abscisic acid response elements (ABRE), drought-induced elements (MBC), and low-temperature response elements (LTR), indicate that *StCLCs* are integral to abiotic stress responses. ABRE elements are linked to ABA-mediated stress signaling, which is critical for salt and drought tolerance (Nakashima and Yamaguchi-Shinozaki, 2013). The presence of MYC-binding elements (G-box) and methyl jasmonate response elements (CGTCA-motif, TGACG-motif) suggests regulation by jasmonic acid (JA), which is known to mediate defense and stress responses in plants (Wasternack and Hause, 2013). These findings align with studies in soybean, where *GsCLC* genes are regulated by ABA and JA under salt stress (Liu et al., 2021; Leung et al., 2023). The diversity of *cis* elements suggests that *StCLCs* are part of a complex regulatory network, enabling potatoes to adapt to multiple abiotic stresses.

The tissue-specific expression analysis (**Figure 9**) revealed high *StCLC* expression in sepals, petals, and roots, with *StCLC3*, *StCLC5*, and *StCLC6* showing elevated expression in roots. This pattern suggests specialized roles in root ion homeostasis, which is critical for nutrient uptake and stress tolerance in potatoes. Roots are primary sites for ion transport, and *CLC* genes in other species, such as soybean *GsCLC-e2*, enhance salt tolerance by regulating Cl^-^ accumulation (Liu et al., 2021). The high expression of *StCLC1* in flowers and *StCLC2* in petals may indicate roles in reproductive development, possibly related to ion balance during pollen tube growth or flower senescence, as reported in Arabidopsis (Song et al., 2009). The differential expression across tissues highlights the functional diversity of *StCLCs* and their involvement in both vegetative and reproductive development.

The qRT-PCR analysis of *StCLC* expression under salt stress (200 mmol/L NaCl; **Figure 10**) revealed dynamic and tissue-specific responses. In roots, the upregulation of *StCLC1*, *StCLC2*, *StCLC3*, *StCLC4*, and *StCLC5* with increasing stress duration, peaking at 48 hours, suggests a robust response to salt stress, likely involving chloride sequestration to mitigate ionic toxicity (Munns and Tester, 2008). *StCLC6* and *StCLC7* exhibited biphasic expression patterns, with peaks at 3 and 48 hours, indicating possible roles in early and late stress responses. In leaves, the expression patterns were more variable, with *StCLC1*, *StCLC2*, and *StCLC5* peaking early (3–12 hours) and declining, while *StCLC3* and *StCLC6* showed sustained increases. *StCLC1*, *StCLC2*, and *StCLC5* show large differences in expression across different tissues under salt treatment at 48 h. This variability may reflect tissue-specific functional requirements, as leaves prioritize photosynthesis and gas exchange, whereas roots focus on ion uptake and exclusion (Shabala and Cuin, 2008). In numerous plant studies, tissue-specific gene expression is a universally observed phenomenon. Under salt stress, the expression levels of certain genes involved in ion transport and osmotic regulation—such as *AtHKT1*—are significantly higher in roots than in leaves. This is because roots, as organs that directly come into contact with soil salts, need to regulate the expression of these genes to maintain intracellular ion homeostasis and reduce the toxic effects of sodium ions (Wang et al., 2018). Although the expression differences of *StCLC1*, *StCLC2*, and *StCLC5* in roots and leaves are different from those in other species, they all reflect the response of the *CLC* gene family to salt stress. The lower expression in leaves compared to roots aligns with findings in longan, where *CLC* genes show tissue-specific stress responses (Liu et al., 2020). The induction of *StCLCs* under salt stress is consistent with patterns in tobacco (*NtCLC* genes; Zhang et al., 2018) and soybean (*GmCLC-g*, *GsCLC*-*g*; Liu et al., 2021), where *CLC* genes are upregulated by salt stress and regulated by ABA and JA signaling pathways (Valenzuela et al., 2016).

The regulation of *StCLC* genes by ABA, JA, and potentially ethylene, as inferred from *cis*-elements and expression patterns, highlights their integration into broader stress signaling networks. ABA is a central regulator of salt stress responses, activating pathways such as the Salt Overly Sensitive (SOS) pathway, which modulates ion homeostasis (Zhu, 2002). In rice, *OsCLC* genes are regulated by ABA-responsive transcription factors like *OsERF* (Ponce et al., 2021), and similar mechanisms may regulate *StCLCs*. The presence of JA-responsive elements suggests that *StCLCs* are also modulated by JA-mediated defense pathways, which are critical for salt tolerance in maize (Ponce et al., 2021). Ethylene, another stress-related hormone, may indirectly influence *StCLC* expression by regulating ion homeostasis, as seen in maize (Ponce et al., 2021). These hormonal interactions suggest that *StCLCs* are part of a multifaceted stress response network, integrating signals from multiple pathways to enhance potato resilience.

The significant induction of *StCLC* genes under salt stress, particularly in roots, suggests their potential as targets for improving salt tolerance in potatoes. Chloride accumulation in roots can prevent toxic ion buildup in shoots, a strategy employed by salt-tolerant plants (Wu and Li, 2019). The dual role of *StCLCs* in chloride and nitrate transport, as inferred from GO analysis, could be leveraged to enhance nutrient uptake efficiency under stress conditions, a critical trait for potato yield stability (Rosales et al., 2020). Overexpression of *CLC* genes, such as *AtCLCc* in Arabidopsis, has been shown to enhance salt tolerance by improving chloride homeostasis (Wei et al., 2016). Similar strategies could be applied to *StCLC3*, *StCLC5*, and *StCLC6*, given their high expression in roots under salt stress. Additionally, the identification of stress-responsive *cis*-elements provides targets for genetic engineering to fine-tune *StCLC* expression under specific environmental conditions.

Plant *CLC* family proteins, acting as chloride channels or transporters, play a core role in maintaining chloride ion (Cl^-^) homeostasis under salt stress. Previous studies have confirmed that the expression level of the *AtCLCf* gene in Arabidopsis thaliana is significantly upregulated by 3-fold in roots after salt treatment; under sodium chloride (NaCl) stress, the *AtCLCf* mutant of the *AtCLCf* gene shows a sensitive phenotype, specifically characterized by shortened root meristematic and elongation zones and a significant reduction in root length, while complemented plants obtained by overexpressing the *AtCLCf* gene can restore the salt tolerance of wild-type Arabidopsis, with the core mechanism being that the *AtCLCf* protein promotes Cl^-^ efflux from roots through Cl^-^/H^+^ antiport activity, reducing Cl^-^ accumulation in the aboveground parts of plants to alleviate ionic toxicity (Rajappa et al., 2024). Evolutionary analysis of the *StCLC* family in potato reveals that members such as *StCLC6* are orthologous to Arabidopsis *AtCLCf*; in addition, potato and tomato belong to the genus *Solanum* in the *Solanaceae* family, sharing high conservation in genome structure, gene family evolution, and stress response mechanisms, which provides a reliable basis for inferring the function of *StCLC* genes using research findings on *SlCLC* genes. Many studies have confirmed that tomato *SlCLC* genes are centrally involved in plant responses to salt stress by regulating Cl^-^ homeostasis (Ma et al., 2025) and combined with the conserved characteristics of gene functions in Solanaceae plants, the key role of *StCLC* genes in potato salt stress adaptation can be confirmed. The cis-elements in the gene promoter region determine the ability of genes to respond to external signals: the promoters of tomato *SlCLC* genes generally contain salt stress-responsive cis-elements such as ABRE (abscisic acid-responsive element), MYB (MYB transcription factor binding site), and MYC (MYC transcription factor binding site), whose presence is directly related to their transcriptional activation characteristics under salt stress (Ma et al., 2025); similarly, the promoter regions of the potato *StCLC* gene family are also enriched with a large number of abiotic stress-responsive elements, including salt stress-related MYB binding sites and hormone-responsive elements, and the activation of these elements is the key regulatory basis for the upregulation of *StCLC* gene expression under salt stress. Combined with transcriptome data and real-time quantitative PCR (qRT-PCR) results, *StCLC* genes exhibit differential expression in different potato tissues, and their expression patterns change under salt stress: in roots, some *StCLC* genes may be rapidly upregulated in the early stage of salt stress, promoting Cl^-^ efflux or transport to aboveground parts to reduce Cl^-^ accumulation in root cells; in leaves, *StCLC* genes may be involved in transporting Cl^-^ to vacuoles to maintain ion balance in mesophyll cells and normal photosynthesis. Integrating research results on the structural characteristics, salt stress response patterns, and regulatory mechanisms of tomato *SlCLC* genes and Arabidopsis *AtCLC* genes, as well as the conservation between potato and tomato in Solanaceae evolution, it can be fully inferred that *StCLC* genes are centrally involved in potato salt stress adaptation by maintaining Cl^-^ homeostasis.

Future studies should focus on functional validation of *StCLC* genes using techniques such as CRISPR/Cas9-mediated knockout or overexpression to elucidate their specific roles in ion transport and stress tolerance. Multi-omics approaches, including transcriptomics, proteomics, and metabolomics, could provide a comprehensive understanding of *StCLC* interactions with other stress-responsive pathways. For example, integrating transcriptomic data with ionomic profiling could clarify the roles of *StCLCs* in chloride and nitrate transport under salt stress. Additionally, exploring the interaction of *StCLCs* with transcription factors, such as MYC or ERF family members, could reveal regulatory mechanisms underlying their stress responses. Comparative studies with other Solanaceae species, such as tomato and eggplant, could further elucidate the evolutionary and functional divergence of *CLC* genes. Finally, field-based studies under varying salinity levels are needed to translate these findings into practical applications for potato breeding, particularly for developing salt-tolerant cultivars suitable for saline soils.

## 5 Conclusions

This study utilized potato genome sequencing data and bioinformatics methods to identify seven members of the potato *StCLC* gene family, each with distinct physicochemical properties. Through phylogenetic analysis, the seven *StCLC* genes were grouped into three clusters, with differences in gene structure between clusters. Colinearity analysis indicated that potato *CLC* genes are more closely related to tomato *CLC* genes. RNA-seq analysis showed that *StCLC3*, *StCLC5*, and *StCLC6* were highly expressed in roots. qRT-PCR results indicated that *StCLC* genes were significantly induced by salt stress. Like other crop *CLC* gene family members, *StCLCs* may actively respond to salt stress and participate in the process of resisting salt stress. Identification and characterization of the *StCLC* gene family provides a foundation for understanding their roles in potato growth, development, and salt stress responses. Their conserved structures, tissue-specific expression, and dynamic regulation under stress highlight their importance in ion homeostasis and abiotic stress tolerance. These insights pave the way for targeted genetic improvements to enhance potato resilience, supporting sustainable agriculture in challenging environments.

## Data Availability

The datasets presented in this study can be found in online repositories. The names of the repository/repositories and accession number(s) can be found in the article/**Supplementary Material**.
